# Fractional CO_2_ Laser versus Fractional Radiofrequency for Skin Striae Treatment: Study Protocol for a Randomized Controlled Trial

**DOI:** 10.3390/healthcare10122372

**Published:** 2022-11-25

**Authors:** Nuno Mendes, Paulo Jorge Alves, Mafalda Barros, Jorge Machado

**Affiliations:** 1 ICBAS—Instituto de Ciências Biomédicas Abel Salazar, Universidade do Porto, 4050-313 Porto, Portugal; 2 CNM—Clínica Nuno Mendes do Grupo Saúde Nuno Mendes, Guilhufe, 4560-164 Penafiel, Portugal; 3 ICS—Instituto de Ciências da Saúde da Universidade Católica Portuguesa—Centro Regional do Porto, 4169-005 Porto, Portugal; 4 CBSin—Center of BioSciences in Integrative Health, 4250-105 Porto, Portugal

**Keywords:** striae, CO_2_, radiofrequency, efficacy, safety

## Abstract

Striae are common dermal lesions associated with physiological and psychological alterations, affecting the quality of life. This proposed randomized controlled trial protocol will evaluate the clinical efficacy and treatment safety of fractional CO_2_ laser versus fractional radiofrequency (FRF) in clinical trials. We will randomly allocate 60 subjects who present abdominal striae into two different groups for the treatment, which will be performed once a month for a 3-month period. The results will be assessed by quartile scoring criteria; by comparing digital photos taken before and after each treatment; and also, by the measurement of cutaneous temperature, skin pH, and elasticity before and after the treatment. This paper describes the rationale and the design of the randomized controlled trial, which may provide evidence for clinical application of the methodology and the quality related to the efficacy and safety of fractional CO_2_ laser versus the FRF protocol for striae treatment.

## 1. Introduction

Striae distensae (SD), also known as stretch marks, affect a large part of the world’s population and are becoming challenging to treat. In 1936, the first correct description of these lesions appeared, entitled as striae atrophicae [1]. Stretch marks are linear prevalent cutaneous lesions [2], characterized by epidermal atrophy [3]. The exact origin of this type of skin condition remains unclear; however, they occur mostly in some physiological and pathological situations, such as in adolescent growth, obesity, pregnancy, and topical steroid use [3,4]. Stretch marks appear at a higher frequency in pregnant woman and adolescents, in whom the prevalence ranges from 6 to 86% [5]. Lower back and knees are the most affected areas in males, while in women they mostly affect the calves, buttocks, and thighs [5].

Clinically, stretch marks are characterized by rapid elongation of the skin, which destroys the elastic fibers, affecting the dermis by decreasing the components of the extracellular matrix (ECM) such as collagen and elastin [5]. In the early stages, SD appear in a pink-red-purple color, with edematous scars called striae rubrae [5,6]. Over time, a decrease in vascularization is observed, with a degradation of collagen and elastin [5], which turns the striae into a white color with an atrophic look, known as striae albae [4,6]. Other different stria types have been identified such as striae caerulea and striae nigrae in individuals with darker skin, which are associated with an increase in their quantity of melanin [7]. Studies also demonstrate that different colors in stretch marks are influenced by melanocytes’ mechanobiology [8].

Despite being a physiological condition, striae still represent a disfiguring condition that creates a lot of psychological problems, affecting the quality of life of any individual with this pathology [9]. Many people look for therapies and treatments, and several have been proposed such as chemical peels, microdermabrasion, topical agents, and ablative and non-ablative energy devices [5]. However, the treatment of SD does not bring 100% effective outcomes since there is no consistent modality available [4]. In the late stages of striae, it becomes even more challenging to find a more effective treatment [2]. One of the modalities that is lately being used to upgrade and recover stretch marks is laser therapy [4], which represents an innovation in the approach to striae, particularly in striae rubrae [10], and is the most common therapeutic alternative recently [11]. Lasers should recover both striae depth and width as well as improve their histology, with an increase in the numbers of collagen and elastin fibers [12].

Skin lasers have improved, even more so after the development of fractional laser treatment, which is characterized by reepithelization due to the migration of viable cells from undamaged sites to the injury site [9]. Laser categories include non-ablative fractional laser, radiofrequency (RF), long pulsed Nd:YAG, fractional CO_2_ laser, fractional bipolar RF, fractional ablative microplasma RF, and intense pulsed light (IPL) [13,14]. Laser therapy represents a great advance in treating stretch marks, specifically striae rubrae, since there is an increase in the vascularization of the lesions [10].

Specifically, fractional CO_2_ laser treatment has been reported as a new resurfacing technique in SD treatments [9]. Here, fractional CO_2_ forms micro-thermal zones of damage, the necrotic debris is expelled, and neo-collagenesis occurs [15]. This technique stimulates epidermal turnover and dermal collagen remodeling, which leads to important improvements in texture and appearance in many types of scars, such as stretch marks [16]—mainly in mature striae [2]. After treatment with fractional CO_2_ laser, full recovery with normal epidermis, parallel arrangement of the collagen bands, and perivascular aggregation of inflammatory cells were demonstrated [16]. In phototype IV skin, the use of CO_2_ laser treatment has been discouraged on account of the high risk of pigmentary alteration [17], although theoretically, this type of laser stimulates fibroblast activity and improves lesions through controlled skin abrasion [18]. Regarding adverse effects, there are a few, and some are self-limiting, such as post-inflammatory hyperpigmentation; however, these effects can be fixed naturally in a few months after treatment [2].

Additionally, CO_2_ laser treatment is more painful and requires a longer recovery time [19] when compared with non-ablative lasers. Studies have shown a significant decrease in the strial surface area after resurfacing with fractional CO_2_ laser treatment when compared with topical therapy (10% glycolic acid + 0.05% tretinoin) [20]. Lee et al. also demonstrated that fractional CO_2_ laser treatment, specifically at 10,600 nm, had a positive effect on late-stage SD, with clinical improvement with only one session [21]. When used in striae albae, fractional CO_2_ laser treatment was shown to be effective in subjects with skin types III and IV [20], and another study also demonstrated that two out of five patients with striae albae showed changes after 4-week treatment intervals with fractional CO_2_ laser [22].

Concerning RF, this type of technique provides different patterns of heat distribution [23]. Unlike lasers, which convert light to heat and focus on specific chromophores, RF devices transfer a high-frequency alternating electrical current that goes through the dermis and hypodermal tissues without affecting the epidermal–dermal barrier [2]. That electrical current is converted into heat, mediating thermal damage to the surrounding connective tissue responsible for denaturing elastic fibers and collagen [24], which occurs immediately after RF treatment [4]. This collagen denaturation represents the tissue contraction, which induces inflammation and stimulates fibroblasts to produce new collagen, new elastin, and ground substances [3]. These alterations intensify the dermal tissue and help to improve the appearance of striae due to the increased production of collagen and elastin [3,24]. Regarding bipolar RF, it has shown clinical and histological improvements in striae distensae, while tripolar RF resulted in a 25–75% improvement in just one week of treatment [25]. Overall, RF is associated with an increase in the production of the dermis, improving SD [26]. A study showed that 14 of 16 subjects observed visible changes, with statistically significant reductions in both the length and width of striae bands [3]. To improve the tissue heating patters, fractional radiofrequency (FRF) was developed, which induces dermal heating [23]. FRF is safe for all skin types due to its “colorblind” nature, being applied directly to the skin surface or within the skin [23]. Even though the side effects were mild and transient, pain remains a significant limitation to the use of FRF, specifically in sensitive body areas such as the abdomen [27].

Overall, only a limited number of studies have been conducted that investigated the effectiveness of different types of treatments for stretch marks, specifically comparing two different types of treatment. Taking that into account and considering the characteristics of both fractional CO_2_ laser and fractional radiofrequency, we would like to focus this work on the evaluation and comparison of the clinical efficacy and treatment safety of both techniques in clinical applications (STRIAL). To evaluate the impact of stretch marks reduction on the quality of life and on body image, two hypotheses are proposed: the clinical efficacy and safety of fractional CO_2_ laser are superior to fractional radiofrequency, or the clinical efficacy and safety of fractional radiofrequency are superior to the fractional CO_2_ laser.

## 2. Materials and Methods

### 2.1. Study Design

**Figure 1:**
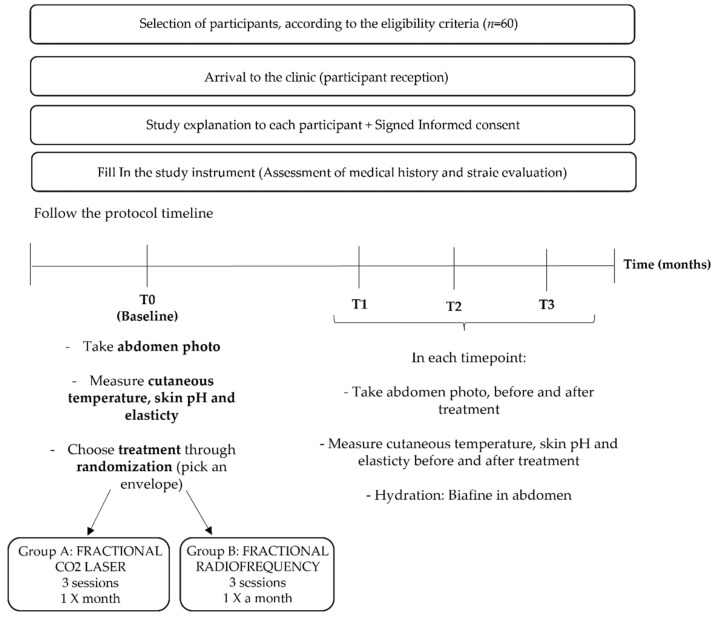
Trial Flow Chart.

We propose a randomized controlled trial, 1 to 1, that aims to compare the clinic efficacy and safety of two different treatments for striae: fractional CO_2_ laser treatment versus fractional radiofrequency (FRF). This trial will be conducted according to the trial procedure presented in the flowchart in Figure 1.

### 2.2. Recruitment and Participants

The flowchart of the study design is shown in Figure 1. The sample will consist of 60 individuals, divided in two main groups: Group A (*n* = 30), receiving fractional CO_2_ laser treatment, and Group B (*n* = 30), receiving fractional radiofrequency treatment. The recruitment and treatment processes will be carried out at Nuno Mendes Clinic in Penafiel, Portugal. First, an advertisement will be posted at the clinic regarding striae treatment for people who have abdominal striae. People who show interest in participating will first be pre-screened and recorded by intake to verify whether they meet the basic inclusion and exclusion criteria. All questions about the study as well as the written informed consent will be explained in detail. All baseline measures should be obtained on a single day.

### 2.3. Eligibility Criteria

Inclusion and exclusion criteria will be applied in the participant selection process. This trial will include male and female patients who are aged 18 years or older and have stretch marks, only in the abdomen area. Patients that refuse to participate in the study; who have a history of keloids, skin infections, or connective tissue disease; or are pregnant will be excluded. Other cases such as participants who take vitamin A and derivatives within two months of the beginning of the study and the use of immunosuppressive drugs will be also excluded from the study.

### 2.4. Allocation

After screening for eligibility and explaining the objectives and procedures for the research project implementation, written informed consent will be obtained from each individual who agrees to participate. In addition, an individual evaluation will be performed regarding the clinical history and the actual striae. Thirty participants will randomly be assigned each to Group A (CO_2_ laser) and Group B (FRF) following a simple randomization procedure involving random selection from two envelopes, each of which will indicate the type of stria treatment.

### 2.5. Intervention

**Figure 2:**
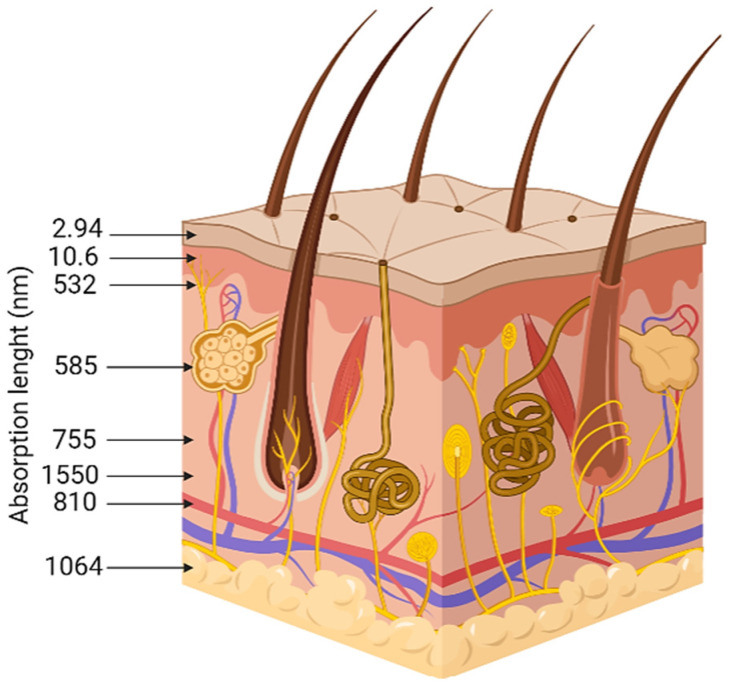
Relative penetration of the most common wavelenght in all layers of the skin. This image was designed based on the literature [29].

**Figure 3:**
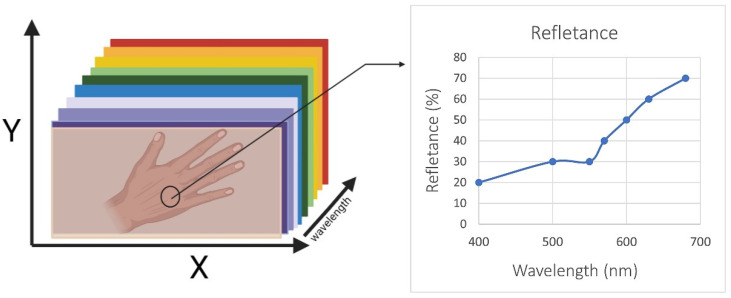
Hyperspectral skin image in the visible light range. This image was designed based on the literature [30].

Regarding the clinical procedures, the CO_2_ laser (CO_2_RE^®^, Candela, Marlborough, MA, USA) group will undergo three sessions, once a month over 3 months. The laser procedure has the following indications: laser tip diameter from 1 to 10 millimeters (mm), according to the width of the lesions; pulse power of 100 W; and repetition rate of 20 Hz using a single pass. During this procedure, a cryotherapy advice (Cryo 6, Zimmer, Germany) will be applied to reduce any pain. Since laser absorption is related to skin properties [28], there is a need to have both the reflectance and absorbance spectra of human skin. This is the key to quantitatively and non-invasively analyzing skin components. Figure 2 represents the relative penetration of different wavelength in all skin layers. Figure 3 represents a hyperspectral skin image, with the reflectance of different wavelength in the visible light range.

The FRF group will also undergo three sessions, once a month over 3 months. The procedure of this treatment will be performed with a set of 24 RF conductive needles. Each needle has a length of 2500 micrometers (μm). Patients will receive initial doses of 20–40 mJ/pin, and the doses will be increased at each session based on patient tolerance. All treated areas will receive a pass based on the treatment site and patient tolerance. During this procedure, cryotherapy will be applied with a Zimmer G system to reduce the patient’s pain.

**Figure 4:**
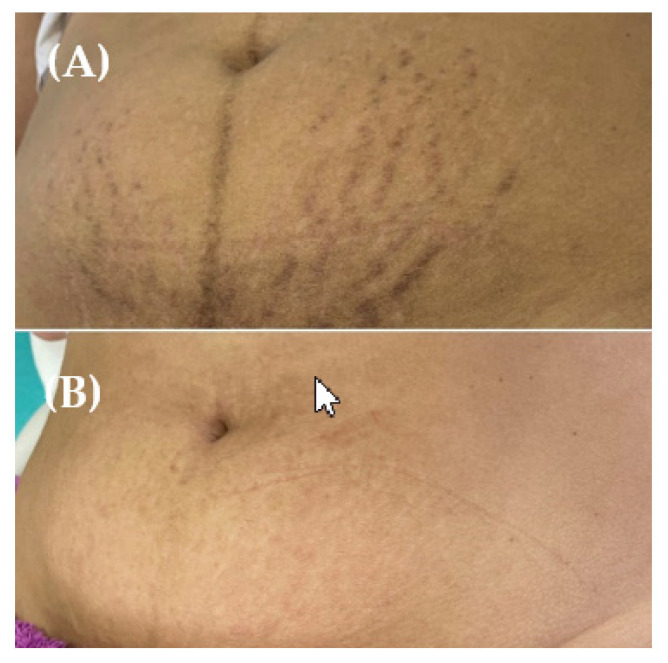
Before (**A**) and after (**B**) control photos of abdominal striae of an individual subjected to laser CO_2_ treatment.

For clinical improvement, before and after photos of an individual that went through laser CO_2_ treatment for abdominal striae will be used as a control (Figure 4); high-resolution digital photographs will be taken before and after both treatments at every session, starting at the baseline. Two physicians blinded to the study will compare these photographs using quartile scoring criteria for the assessment (0 = no improvement; 1 = mild (25% improvement); 2 = moderate (>25 ≤ 50% improvement); 3 = good (>50 ≤ 75% improvement); 4 = excellent (≥76% improvement)).

Additionally, measurements of cutaneous temperature, pH, and skin elasticity will be taken at the baseline and before and after each treatment. Cutaneous temperature will be measured by a thermograph (FLIR i7), positioned at 7 cm away from the abdomen area. The skin pH will be measured by pH bands. The skin elasticity will be measured by an elastometer.

## 3. Results

### 3.1. Primary Outcome

The primary outcome measure will focus on the change in the quartile scoring criteria by comparing the digital photos taken before and after each treatment (fractional CO_2_ laser and FRF) for clinical improvements in the affected area (abdomen). This process will be assessed by two physicians blinded to the study. Figure 4 will be the control, being compared to the pictures taken during the study.

### 3.2. Secondary Outcome

Secondary outcome measures will focus on data changes regarding cutaneous temperature and skin pH and elasticity, assessed after the stria treatments (fractional CO_2_ laser versus FRF). Cutaneous temperature will be measured by a thermograph (FLIR E1), which will help to evaluate any inflammatory process; skin pH will be measured by pH bands; and skin elasticity will be measured by an elastometer.

### 3.3. Participant Timeline

The timeline of the study visits, enrolment process, interventions, and assessments carried out on the participants will be reported according to the Standard Protocol Items: Recommendations for Interventional Trials (SPRIT) guidelines [31], as shown in Table 1.

### 3.4. Adverse Events

Adverse events will be monitored for each treatment during the trial. Any adverse events or reactions that are thought to be causally associated with the intervention, such as discomfort, mild pain, tissue erythema, tissue edema, irritation, and hyperpigmentation or hypopigmentation of the tissue, will be reported and managed. The measurement of cutaneous temperature before and after the treatments will help us monitor any sudden and significant changes regarding inflammatory processes. If the individual performs any additional physical therapy a day before or 3 days after the treatment, it will also increase the possibility of erythema and irritation since heat is being applied to the skin, so it is not advisable to perform exercise during this time.

### 3.5. Data Collection and Statistical Analysis

Data collection will be performed in every session for each treatment. The data from this study will be analyzed using the Statistical Package for the Social Sciences (SPSS) V.21 software. A *p*-value < 0.05 will be considered statistically significant. Measured data will be expressed as mean ± SD.

## 4. Discussion

As we mentioned before, from a previous review of the literature, we concluded that for an accurate assessment of efficient treatments for striae, more clinical studies with a significant sample are necessary. Therefore, our trial seems to be an innovative study exploring the influence of stria treatment on the health-related quality of life of patients undergoing fractional CO_2_ laser or fractional radiofrequency treatment. To overcome some limitations found in the literature, we propose a randomized controlled trial with a sample of 60 individuals. This sample would be important to increase the probability of including both men and women and participants with different types of skin and strial bands.

With the proposed trial, we expect to validate the fractional CO_2_ laser and fractional radiofrequency protocols in terms of efficacy and safety, which can be useful in the management of physical characteristics linked to striae. With this study design, we expect to find evidence that the treatments could demonstrate significant differences, such as a reduction in the length and width of striae bands. With respect to the primary outcomes, we will explore the quartile scoring criteria prepared for the assessment of clinical improvements in the affected area (abdomen) before and after the respective treatments, and we expect to see differences by comparing the digital photos taken in these timelines, using Figure 4 as the control. Regarding the secondary outcomes, we will explore the measurements of cutaneous temperature, skin pH, and skin elasticity to assess any inflammatory processes and to describe differences in different skin aspects after each treatment.

In addition, we will try to assess the impact of stretch mark reduction on body image, through a scale that evaluates the quality of life, the BODY-Q^TM^ Scale—Appraisal of Stretchmarks. 

## 5. Conclusions

In conclusion, this study protocol offers a randomization procedure for our subsequent clinical research. We expect that the results will provide evidence on the effectiveness and safety of fractional CO_2_ laser and fractional radiofrequency treatments by comparing the differences of these two treatments on the abdomen area, as well as suggestions for further research.

## Figures and Tables

**Table 1:** Timing of visits and data collection, according to the Standard Protocol Items: Recommendations for Interventional Trials (SPIRIT).

				Treatment Period		
		Screening	Baseline 0-Month	1-Month	2-Month	3-Month
Patient	Eligibility Informed consent Clinical data Physical examination Randomization	X	X X X X			
Intervention	Fractional CO_2_ laser: Group A			1 session per month for 3 months		
	Fractional radiofrequency: Group B			1 session per month for 3 months		
Outcomes	Digital photo collection		X	X	X	X
	Cutaneous temperature		X	X	X	X
	Skin pH		X	X	X	X
	Skin elasticity		X	X	X	X
Participant Safety	Adverse effects			X	X	X

## Data Availability

Not applicable.
